# In vitro comparison of peracetic acid and autoclave sterilization in the corrosion of orthodontic pliers: a pilot study

**DOI:** 10.1590/2177-6709.26.5.e2119353.oar

**Published:** 2021-10-25

**Authors:** Livia Kelly Ferraz NUNES, Érika Lopes CARNEIRO, Nathalia Barbosa PALOMARES, Lilian SENNA, Helio SAMPAIO-FILHO, José Augusto Mendes MIGUEL

**Affiliations:** 1 Universidade do Estado do Rio de Janeiro, Faculdade de Odontologia, Departamento de Ortodontia (Rio de Janeiro/RJ, Brazil).; 2 Private practice (Rio de Janeiro/RJ, Brazil).; 3 Universidade do Estado do Rio de Janeiro, Departamento de Química Analítica (Rio de Janeiro/RJ, Brazil).; 4 Universidade do Estado do Rio de Janeiro, Departamento de Prótese Dentária (Rio de Janeiro/RJ, Brazil).; 5 Universidade do Estado do Rio de Janeiro, Faculdade de Odontologia, Departamento de Ortodontia (Rio de Janeiro/RJ, Brazil).

**Keywords:** Sterilization, Corrosion, Autoclave, Peracetic acid

## Abstract

**Introduction:**

The most currently recommended method for sterilization of orthodontic pliers is the autoclave, while peracetic acid has also been shown to be effective in the chemical sterilization process.

**Objective:**

This study sought to compare the corrosive effects of peracetic acid and autoclave sterilization process of orthodontic pliers.

**Methods:**

Four active tungsten carbide (WC) stainless steel tie-cutting pliers from the manufacturers Quinelato (Rio Claro, SP, Brazil) and ICE (Cajamar, SP, Brazil) were selected. The active ends of the pliers were sectioned, and six active tips were obtained and distributed into the following groups: 1) control group (no sterilization); 2) AC group (two active pliers tips submitted to 100 autoclave sterilization cycles); and 3) AP group (two active pliers tips submitted to 100 cycles of sterilization by immersion in 2% peracetic acid solution for 30 minutes).

**Results:**

Chemical analysis using X-ray dispersive energy spectroscopy showed that after autoclave sterilization, only the ICE pliers presented oxidation corrosion (Δ[O] = +24.5%; Δ[Fe] = +5.8%; Δ[WC] = -1.9%). In comparison, following peracetic acid sterilization, both manufacturers ICE (Δ[O] = +1.8%; Δ[Fe] = +18.0%; Δ[WC] = -1.1%) and Quinelato (Δ[O] = +5.3%; Δ[Fe] = -10.4%; Δ[WC] = -15.2%) showed corrosion. The morphological analysis revealed that peracetic acid caused a pitting and localized corrosion in both brands, while the autoclave caused uniform surface corrosion on the ICE pliers.

**Conclusion:**

Autoclave application was the sterilization method that generated less corrosive damage to the orthodontic cutting pliers, when compared to the immersion in 2% peracetic acid.

## RESUMO

**Introdução:**

O método mais recomendado para a esterilização dos alicates ortodônticos é a autoclave; porém, o ácido peracético mostrou-se eficaz no processo de esterilização química.

**Objetivo:**

O objetivo do presente estudo foi avaliar o efeito corrosivo do ácido peracético e da autoclave no processo de esterilização de alicates ortodônticos.

**Métodos:**

Foram selecionados quatro alicates de corte de amarrilho de aço inoxidável, com ponta ativa de carboneto de tungstênio (WC), das marcas Quinelato (Rio Claro/SP, Brasil) e ICE (Cajamar/SP, Brasil), que foram distribuídos em três grupos: 1) Controle (C), o qual não foi submetido à esterilização; 2) Grupo AC, constituído por duas pontas ativas de alicates submetidas a 100 ciclos de esterilização em autoclave; e 3) Grupo AP, formado por duas pontas ativas de alicates submetidas a 100 ciclos de esterilização por imersão em solução de ácido peracético a 2% durante 30 minutos.

**Resultados:**

Por meio da análise química (EDS, energia dispersiva de raios X), constatou-se que, após esterilização em autoclave, somente o alicate ICE apresentou corrosão por oxidação (Δ[O] = +24,5%; Δ[Fe] = +5,8% e Δ[WC] = -1,9%), enquanto na esterilização em ácido peracético, ambas as marcas, ICE (Δ[O] = +1,8%; Δ[Fe] = +18,0% e Δ[WC] = -1,1%) e Quinelato (Δ[O] = +5,3%; Δ[Fe] = -10,4% e Δ[WC] = -15,2%), apresentaram corrosão. A análise morfológica demonstrou que o ácido peracético causou uma corrosão localizada em pite em ambas as marcas, enquanto a autoclave ocasionou uma corrosão superficial uniforme no alicate ICE.

**Conclusão:**

A autoclave foi o método de esterilização que gerou menor dano corrosivo aos alicates ortodônticos, em comparação à imersão em ácido peracético a 2%.

## INTRODUCTION

According to biosafety principles, the sterilization of orthodontic tools is essential to prevent cross-infection among patients, mainly due to saliva or blood contamination. Although there are several chemical and physical sterilization methods, the autoclave is the most widely used and recommended for dental use.^1^

During the autoclave sterilization process, the materials undergo combined actions of temperature and humidity exposure. The steam moistens the materials, transmit heat, pressure and eliminates microorganisms, bu means of protein coagulation.^2^ Nevertheless, repeated cycles of autoclave sterilization can induce corrosion on metallic instruments,^3^ and may negatively influence the ability of the instruments subjected to such a process, especially cutting pliers.^4^ These damages could lead to a reduction in the duration of the pliers and an increase of cost, due to the need of replacing these orthodontic materials.

Specifically in cutting pliers, the corrosion affects three areas: the hinge, the cutting blade and the blade weld joint. This damage ranges from uniform corrosion (which affects the entire surface area, with less harm) to pitting localized corrosion (that is more destructive and involves the formation of small cavities on the surface).^5^ However, both types of corrosion can lead to loss of cutting efficiency.^4^

Although the damage to orthodontic pliers may result from several etiologies, such as improper use and mechanical fatigue, the most frequent cause is autoclave sterilization, due to the combination of heat and humidity.^3^

Among the new options for sterilization methods, the peracetic acid stands out, which has been proven effective in the disinfection and sterilization of dental materials.^6^ ^,^ ^7^ This is a high-level disinfectant that induces protein denaturation and breakdown of cell-membrane permeability.^1^

Few studies analyzed the corrosive effects of 2% peracetic acid solution in orthodontic materials. In 2016, Tavares^8^ demonstrated that a 2% peracetic acid solution was effective in the chemical sterilization of pliers, bands, and elastic bands, as it eliminated 100% of microorganisms after 15 minutes of immersion. Whereas, in the study by Wichelhaus et al,^5^ the results indicated that peracetic acid sterilization caused significantly less surface corrosion than the heat sterilization method. However, since 2012, the use of dry sterilization (heat sterilization method) has been outlawed by the Brazilian Health Regulatory Agency (ANVISA) for sterilization of health products, according to the RDC no. 15/2012.

Thus, there is a need for new studies that evaluate the corrosion effect of these two different sterilization processes over orthodontic ligature pliers. In this study, therefore, we considered the evaluation of the exposure of the pliers to 2% peracetic acid (chemical) and the traditional autoclave (saturated pressure steam).

## MATERIAL AND METHODS

### SAMPLE

Four orthodontic tie-cutting pliers from two different manufacturers (Lot 16065; Quinelato, Rio Claro/SP, Brazil; and Lot 111604; ICE, Cajamar/SP, Brazil) were selected. All cutting pliers used consisted of stainless steel and active widia tip (tungsten carbide) as factory-made.

### PREPARATION OF THE SPECIMENS

The active ends of the four orthodontic cutting pliers were equally sectioned at the plier junction, with a carborundum disc, coupled to a straight and low-rotation engine. Thus, eight active tips were obtained. To create the study specimens, six of the eight active tips were selected and embedded in a self-curing epoxy resin (Aka-Resin Liquid Epoxy; Akasel A/S, Roskilde, Denmark).

Subsequently, the six specimens were subjected to metallographic preparation in accordance with the American Society for Testing and Materials E 883-86 standard, to remove any porosity or irregularity from the specimens. The specimens were polished using a Politriz machine (APL-4; Arotec, Cotia/SP, Brazil), with the sequence of #400, #600, #1200, #1500, and #2000 granulation sandpaper (Arotec, Cotia/SP, Brazil) and finishing with felt associated with diamond paste.

### STERILIZATION METHODS USED IN THE SAMPLE

Three groups were analyzed, as follows:

1) A control group, consisting of two samples of plier tips, which were not submitted to any kind of sterilization.
2) AC group, composed of two samples of plier tips, submitted to 100 autoclave sterilization cycles (Vitalle 21L; Cristófoli, Paraná, Brazil) in the sterilization sector of the Orthodontics Department of the UERJ School of Dentistry. The pliers were sealed inside self-sealing envelopes (Amcor, Cambé, Paraná, Brazil) and autoclaved during a 45-minute sterilization and drying cycle at 132°C and 1 atm pressure. After each cycle, the pliers were removed from the self-sealing envelope, sealed in a new envelope and reinserted into the autoclave.
3) AP group, formed by two samples of plier tips, submitted to 100 cycles in 2% peracetic acid (Sekusept Aktiv; Ecolab, St. Paul, MN, USA) for 30 minutes. The samples were then washed in sterile distilled water, to remove any corrosive residue, and dried with disposable paper towels.

### MICROSCOPIC ANALYSIS OF CORROSIVE EFFECTS OF STERILIZATION PROCESSES

Upon completion of the sterilization cycles, the control, AC, and AP groups were subjected to microscopic analysis at the Microscopy Laboratory of the Fluminense Federal University School of Dentistry (Nova Friburgo/RJ, Brazil). For the morphological analysis of corrosion, a region of interest was determined at a point 3 mm from the active tip of the pliers, which is the most important functional part of the instrument. All of the samples were submitted to morphological analysis via scanning electron microscopy (SEM) (model JSM-IT300LVl JEOL Ltd., Tokyo, Japan) under 500× magnification.

In addition, X-ray dispersive energy spectroscopy (EDX) was performed, which analyzes surfaces of different structures by means of chemical micro-ray analysis. This microanalysis enabled the elucidation of the metallic composition of the pliers as factory standard (control group), and also the detection of changes in the chemical composition of the pliers’ tips submitted to the two tested sterilization methods (groups AC and AP).

## RESULTS

### CONTROL GROUP

**Figure 1:**
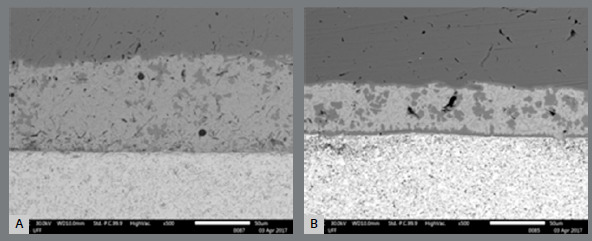
Photomicrographs of the active tips of the control group pliers, at 500× magnification during SEM: A) ICE plier; B) Quinelato plier.

The photomicrographs of the active tips, obtained under 500× magnification during SEM for morphological analysis of the pliers as factory standard, are shown in Figure 1.

**Figure 2:**
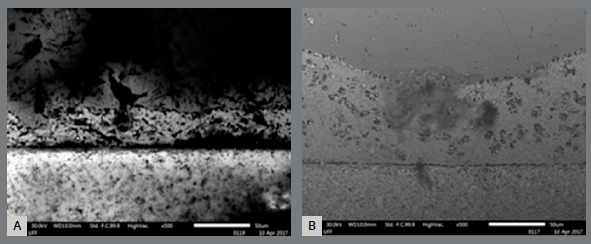
Photomicrographs of the active tips of the autoclave group pliers, at 500× magnification during SEM: A) ICE plier; B) Quinelato plier.

**Figure 3:**
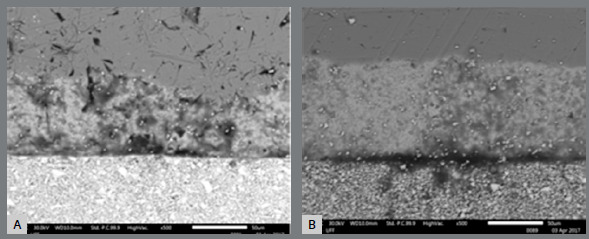
Photomicrographs of the active tips of the peracetic group pliers, at 500× magnification during SEM: A) ICE plier; B) Quinelato plier.

The active tip of the cutting pliers of both manufacturers demonstrated three components on their active tip (the numbers below correspond to the regions identified in the photomicrographs of Figures 1, 2, and 3), as follows:

1) Stainless steel region, mainly composed by an alloy of iron (Fe) and chromium (Cr), but with other metals, such as nickel (Ni) and carbon (C).
2) Silver weld, which is composed of an alloy of silver (Ag), copper (Cu), and zinc (Zn), commonly including other metals, such as manganese (Mn) and tin (Sn).
3) Cutting blade, which is mainly composed of tungsten carbide (WC), with a very low content of other components, such as cobalt (Co).

The results of the EDX chemical analysis are presented in Table 1.

**Table 1:** Superficial chemical microanalysis of the alloys present in the active end of the control group of ICE and Quinelato pliers, obtained by the EDX analysis of SEM.

CHEMICAL ELEMENTS	ICE	QUINELATO
	Control (wt.%)	Control (wt.%)
Fe	18.6%	26.8%
Cr	3.6%	5.2%
C	-	7.0%
Ag	24.4%	5.4%
Zn	9.0%	1.9%
Cu	6.6%	1.6%
Mn	3.0%	1.0%
Al	0.7%	0.5%
Ni	2.1%	0.7%
WC	27.5%	40.5%
Co	-	6.4%
O	4.4%	3.1%

In the region of stainless steel, the Quinelato pliers presented a composition consisting of 26.8% Fe, 5.2% Cr, 7% C, and 0.7% Ni. For the ICE pliers, the percentages of Fe (18.6%) and Cr (3.6%) were lower, and the Ni (2.1%) was higher.

In the silver solder region, all of the constituent chemical elements presented higher percentages in the ICE pliers than the Quinelato ones.

In the cutting region, a higher percentage of WC was detected in the Quinelato pliers in relation to the ICE ones. Furthermore, for both manufacturers, a heterogeneous surface was observed and showed compacted WC grains held together by a Co matrix; some grains were not covered by the matrix. The ICE pliers presented an even more heterogeneous surface, without a Co matrix in its composition.

Both brands presented the presence of oxygen (O), which causes oxidation to the material, with higher concentrations in ICE pliers (O = 4.4%) than in Quinelato ones (O = 3.1%).

### AUTOCLAVE GROUP

Photomicrographs of the active tips at a 500× magnification taken during SEM are presented in Figure 2. The results of the EDX analysis of the alloys presented in the autoclave active group of ICE and Quinelato pliers are presented in Tables 2 and 4.

**Table 2:** Superficial chemical microanalysis of the alloys present in the active end of the autoclave group of ICE and Quinelato pliers, obtained by the EDX analysis of SEM.

Chemical elements (%)	ICE		QUINELATO	
	Control	100 cycles Peracetic acid	Control	100 cycles Peracetic acid
Fe	18.6%	24.4%	26.8%	31.8%
Cr	3.6%	5.1%	5.2%	6.9%
C	-	-	7.0%	-
Ag	24.4%	9.6%	5.4%	24.3%
Zn	9.0%	1.0%	1.9%	5.7%
Cu	6.6%	1.8%	1.6%	6.9%
Mn	3.0%	0.4%	1.0%	1.3%
Al	0.7%	0.9%	0.5%	-
Ni	2.1%	-	0.7%	-
WC	27.5%	25.6%	40.5%	21.2%
Co	-	1.8%	6.4%	-
O	4.4%	28.9%	3.1%	-

**Table 3:** Superficial chemical microanalysis of the alloys present in the active end of the peracetic group of ICE and Quinelato pliers, obtained by the EDX analysis of SEM.

Chemical elements (%)	ICE		QUINELATO	
	Control	100 cycles Peracetic acid	Control	100 cycles Peracetic acid
Fe	18.6%	36.6%	26.8%	16.4%
Cr	3.6%	6.8%	5.2%	3.3%
C	-	-	7.0%	-
Ag	24.4%	11.2%	5.4%	23.5%
Zn	9.0%	4.2%	1.9%	8.3%
Cu	6.6%	3.1%	1.6%	6.3%
Mn	3.0%	1.1%	1.0%	2.7%
Al	0.7%	1.1%	0.5%	-
Ni	2.1%	1.3%	0.7%	1.8%
WC	27.5%	26.4%	40.5%	25.3%
Co	-	-	6.4%	2.3%
O	4.4%	6.2%	3.1%	8.4%

**Table 4:** Comparison of the superficial chemical microanalysis of the alloys present in the active ends of the ICE and Quinelato pliers, in the control, AP and AC groups, obtained by the EDX analysis of SEM.

Chemical elements (%)	ICE					Quinelato				
	Control (C)	Group AP	Δ AP-C	Group AC	Δ AC-C	Control (C)	Group AP	Δ AP-C	Group AC	Δ AC-C
Fe	18.6	36.6	+ 18.0	24.4	+5.8	26.8	16.4	-10.4	31.8	+5.0
Cr	3.6	6.8	+3.2	5.1	+1.5	5.2	3.3	-1.9	6.9	+1.7
C	-	-	-	-	-	7.0	-	-7.0	-	-7.0
Ag	24.4	11.2	-13.2	9.6	-14.8	5.4	23.5	+18.1	24.3	18.9
Zn	9.0	4.2	-4.8	1.0	-8.0	1.9	8.3	+6.4	5.7	3.8
Cu	6.6	3.1	-3.5	1.8	-4.8	1.6	6.3	+4.7	6.9	+5.3
Mn	3.0	1.1	-1.9	0.4	-2.6	1.0	2.7	+1.7	1.3	+0.3
Al	0.7	1.1	+0.4	0.9	+0.2	0.5	-	-0.5	-	-0.5
Ni	2.1	1.3	-0.8	-	-2.1	0.7	1.8	+0.9	-	-0.7
WC	27.5	26.4	-1.1	25.6	-1.9	40.5	25.3	-15.2	21.2	-19.3
Co	-	-	-	1.8	+1.8	6.4	2.3	-4.1	-	-6.4
O	4.4	6.2	+1.8	28.9	+24.5	3.1	8.4	+5.3	-	-3.1

The ICE pliers showed higher concentrations of Fe, Cr, and O in the stainless-steel region, when compared with the control group. This finding demonstrates a degree of surface corrosion, with surface oxide formation (Cr and/or ferrous oxide). In comparison, the Quinelato pliers showed no corrosion, since the presence of O was not detected after 100 autoclave cycles.

In the silver weld region of the ICE pliers, all of the chemical elements exhibited lower percentages in the autoclave group than in the control group. For the Quinelato pliers, the opposite was detected; lower concentrations in the control group were observed, when compared with the autoclave test group.

In the cutting region, lower WC concentrations were detected in the pliers of both manufacturers submitted to autoclave sterilization, although a more significant degree was found in the Quinelato pliers than in the ICE pliers, suggesting that carbide crystals were released into the medium of WC. In addition, the matrix that surrounds the Quinelato brand WC was completely lost after 100 autoclaving cycles.

### PERACETIC ACID GROUP

The photomicrographs of the active tips obtained at a 500× magnification in the SEM are shown in Figure 3. The EDX analysis of the alloys present in the active tips of the peracetic acid group of the manufacturers ICE and Quinelato is presented in Table 3 and 4.

After 100 cycles immersed in peracetic acid, it could be noticed oxidation in the stainless steel region of the ICE pliers, with higher concentrations of Fe, Cr, and O (indicating oxidation, in comparison to the control group). In the Quinelato pliers, oxidation was noticed due to an increase in the percentage of O (not because Fe and Cr concentrations).

In the silver solder region, all chemical elements that form this area showed a reduction in the ICE pliers. Conversely, in the Quinelato pliers there was an increase, indicating that oxidation occurred in this region.

In the cutting region, there was a percentage loss of the WC in both brands; however, the Co matrix, present only in the Quinelato brand, was not completely eliminated after peracetic acid immersion.

## DISCUSSION

To minimize, prevent, or reduce the risk of cross-infection between patients within the orthodontic office, standard precautionary measures should be adopted in daily practice.^1^ ^,^ ^9^ ^-^ ^11^

Many orthodontists neglect to pursue optimal infection control, and end up performing only material disinfection.^12^ The reasons for the high percentage of disinfection noncompliance can be attributed to the large volume of patients treated on a day, the shorter duration of care, the cost of care, time taken for the entire sterilization process, and the shortening of the duration of the pliers when subjected to constant autoclave sterilization.^3^

In the orthodontic office, orthodontic pliers are the most-used instruments during patient care.^13^ ^,^ ^14^ In the present research, orthodontic tie-cutting pliers were chosen because of the nature of the activity and sensitiveness to damage at the cutting active tip.^5^ ^,^ ^15^ Thus, four orthodontic tie-cutting pliers from two different manufacturers were tested (ICE and Quinelato). The small sample size was enough to detect differences between the groups, because of the *in-vitro* setting. Only the active tips of these pliers were evaluated, as the active tip is the most important functional part of the cutting pliers.

Corrosion depends on the material, the corrosive medium and the process conditions to occur.^16^ Therefore, it is important to verify the effect of both disinfection and sterilization processes in the corrosion of the pliers. Meanwhile, as the autoclave is considered the safest and recommended sterilization process because it eliminates all life forms,^1^ this method was used as the gold standard in the present study. Some authors^3^ ^,^ ^15^ ^,^ ^17^ ^,^ ^18^ stated that the humid heat of the autoclave can cause pliers to corrode. However, Jones et al^19^ used a visual scale to analyze orthodontic pliers after six months of autoclave sterilization, and found satisfactory results, corroborating the outcomes of other study^20^ that did not detect any deterioration in the active tip of the pliers after autoclave and heat sterilization cycles. However, it is noteworthy that Jones et al^19^ used an ineffective method of evaluation, and Vendrell et al^20^ performed only a few sterilization cycles (only 6 and 12 cycles), making their findings questionable.

Still, these prior results partially corroborate the data obtained by the present study, where, following 100 autoclave cycles, it was found corrosion in the pliers of only one of the manufacturers (ICE). No corrosion was detected in the Quinelato pliers. These data can be explained by the percentage differences in the alloys used to manufacture the Quinelato and ICE pliers, as observed in the control group.

As an alternative method to autoclave use, the most widely accepted chemical agent for instrument disinfection is 2% peracetic acid.^1^ This agent demonstrates excellent efficacy; easy to use; noncorrosive; patient/professional/environmental safety; and is nontoxic and nonallergenic at low concentrations. 

Several studies to date confirmed the efficacy of this chemical disinfectant and sterilizer,^21^ ^,^ ^22^ although few evaluated its corrosive effect on dental materials. In orthodontics, Wichelhaus et al^5^ evaluated distal and Weingart orthodontic pliers after 500 oven sterilization cycles and 5% peracetic acid. After evaluation under SEM, researchers concluded that peracetic acid caused less corrosion than the heat sterilization method. However, the type of corrosion caused by peracetic acid (pitting corrosion) was considered more harmful than that generated by the heat sterilization (uniform). The results of the present study corroborate these previous data, as 2% peracetic acid was corrosive for both manufacturers, but less corrosive for the ICE pliers.

For better corrosion assessment, it is necessary that the alloy composition of each pliers’ manufacturer be evaluated by EDX-type SEM analysis. The literature presents only one study conducted with this methodology. Benyahia et al^4^ analyzed the corrosion of orthodontic cutting pliers from different manufacturers after 50 autoclave cycles and 50 cycles in different chemical disinfectants (peridiol E 1%; hexanios G + R 0.5%; steranios 2%). Corrosion manifested differently depending on the sterilization method used, and chemical disinfection was more aggressive than autoclave sterilization. The data from the present study corroborate the results of Benyahia et al,^4^ since autoclave caused corrosion in the pliers of only one manufacturer (ICE), while 2% peracetic acid generated corrosion in both brands (ICE and Quinelato).

In the present study, Quinelato cutting pliers presented in the region of stainless steel percentages of Fe (26.8%), Cr (5.2%), C (7%), and Ni (0.7%). In turn, the ICE pliers showed a lower percentage of Fe (18.6%) and Cr (3.6%), while Ni (2.1%) was at a higher percentage.

Cr is one of the most important elements in the composition of the pliers, as it provides excellent corrosion resistance to the material, being responsible for the formation of a surface protective film that drastically reduces the corrosion rate. This protective film is formed when Cr in contact with O oxidizes, forming a thin and stable layer of chromium oxide (Cr_2_O_3_). This prevents O from flowing into the steel and protects against corrosive processes.

The chemical element C, which was found only in the Quinelato pliers, is added to the pliers’ composition to increase the hardness of the material. Ni improves alloy strength at high temperatures and provides ductility and weldability, improving overall strength. Therefore, as the Quinelato pliers presented C and a higher Cr content, it is reasonable that these pliers did not corrode after 100 autoclave sterilization cycles. In a different way, corrosion was detected in the ICE pliers after the same procedure.

The silver solder, which presents in its composition Ag, Cu, Zn, and Mn, allows for the union between the metals that compose the pliers. After 100 cycles in peracetic acid, the ICE pliers presented a reduction of all chemical elements of silver solder, with the loss of these elements occurring to the medium in which they were immersed. However, there was an increase of these components in the Quinelato pliers, together with an increase of O, which indicates that oxidation occurred in this region after sterilization in peracetic acid.

Widia is a cluster of rare metal carbides (mainly tungsten, tantalum, titanium, and molybdenum) with a cobalt or nickel binder, in which the cohesion of the assembly is performed by sintering. As it is very resistant to mechanical wear, Widia is generally used on the active cutting part, being welded or attached to the body of the tool. In addition, WC is surrounded by a matrix, commonly of low-grade cobalt element (Co), which fills the voids between the carbide grains. After 100 cycles in autoclave and 2% peracetic acid exposure, a decrease in this component in both brands occurred, along with a decrease in the Quinelato Co matrix, indicating a loss of this component.

The results of this research show that peracetic acid was the most harmful to the pliers of both manufacturers, as it generated an outcome of localized pitting corrosion, while the autoclave caused uniform surface corrosion only in the ICE pliers.

Uniform corrosion is less harmful than localized corrosion^5^ and can be eliminated by mechanical removal (polishing)^5^. Therefore, this result confirms that the autoclave is the most recommended sterilization method, corroborating ANVISA’s recommendation that metallic materials should be sterilized by a physical process, as they are heat-resistant. Chemical sterilization should be used on heat-sensitive materials only if there is no other method to replace it.^1^

The present study presents key unpublished results on the comparison of the corrosive effects of peracetic acid and the autoclave, despite the small number of samples. It is noteworthy that the clinical use of the pliers may exhibit different results, as other factors - such as mechanical fatigue, blood or saliva residues, cleaning and rinsing without distilled water, improper drying, and lack of lubrication - can also lead to corrosion of pliers.^4^ ^,^ ^19^ ^,^ ^20^ Therefore, future research should also test pliers after clinical use and sterilization protocols for each method evaluated, following the manufacturer’s recommendations for disinfection, drying, lubrication, packaging, sterilization, and storage of orthodontic cutting pliers.

## CONCLUSION

From the results of this pilot study, it can be concluded that both sterilization methods caused corrosion to the pliers. However, sterilization by immersion in 2% peracetic acid seems to have produced more harmful corrosion when compared with the autoclave method.

Importantly, the development of new research including the evaluation of corrosion after clinical use is essential to expand the knowledge on this subject and the evaluation of other factors related to corrosion.
